# The prognostic significance of primary tumor laterality in malignant ovarian teratomas: a 10-year experience at a single institution

**DOI:** 10.3389/fimmu.2025.1700779

**Published:** 2026-01-09

**Authors:** Xuechao Ji, Zian Zheng, Jing Yang, Guoliang Li, Xiangyu Liu

**Affiliations:** 1 Department of Obstetrics and Gynecology, The Affiliated Hospital of Qingdao University, Qingdao, Shandong, China; 2 Department of Gynecology, Weifang People’s Hospital, Shandong Second Medical University, Weifang, Shandong, China; 3 Department of Medical Administration, The Affiliated Hospital of Qingdao University, Qingdao, Shandong, China; 4 Department of Radiation Oncology, The Affiliated Hospital of Qingdao University, Qingdao, Shandong, China

**Keywords:** immature teratoma, malignant ovarian teratoma, malignant transformation of mature cystic teratoma, primary tumor laterality, prognosis

## Abstract

**Background:**

Approximately 95% of ovarian teratomas are classified as benign, with only about 5% being malignant. Nevertheless, no research has previously explored the connection between primary tumor laterality and prognostic outcomes among malignant ovarian teratoma (MOT) patients. Our aim was to investigate the association of primary tumor laterality with prognosis in MOT.

**Methods:**

This retrospective study enrolled patients with MOT from The Affiliated Hospital of Qingdao University from January 2012 to December 2021. The primary outcome was progression-free survival (PFS) and overall survival (OS). The prognostic difference between left-sided, right-sided and bilateral groups was investigated using Kaplan-Meier analyses and Cox proportional hazards regression analyses.

**Results:**

A total of 51 eligible patients with MOT were included with a median age of 52 years. Among the patients with MOT, the multivariate Cox regression analyses showed that right-sided (hazard ratio [HR]=0.01; 95% confidence interval [CI]=0.00-0.04; *P* = 0.01) MOT was associated with better PFS, compared with left-sided MOT (HR = 1.00). Kaplan-Meier analyses also showed that the primary tumor laterality had a significant prognostic effect in MOT.

**Conclusion:**

Among patients with MOT, those with unilateral tumors, particularly right-sided ones, had a significantly better prognosis than those with bilateral tumors. Gynecologic oncologists might account for the prognostic impact of primary tumor laterality in MOT and tailor treatment and surveillance accordingly.

## 1 Background

Ovarian teratomas originate from the primitive germ cells found in the embryonic ovary. This type of tumor represents the most prevalent category of ovarian germ cell tumors, accounting for 36% to 69% of cases (1, 2). Approximately 95% of ovarian teratomas are classified as benign, with only about 5% being malignant (3). Mature cystic teratomas are the most common type of ovarian tumor in individuals of reproductive age, comprising tissues derived from the ectoderm, mesoderm, and endoderm (4). Although malignant ovarian teratomas (MOT) are infrequent in clinical settings, their incidence ranges from 0.2 to 0.5 per 100,000 (5). The clinical signs of MOT are often indistinguishable from those of benign teratomas (6, 7). Only a limited number of MOT cases can be identified prior to surgery, creating challenges for gynecologists and oncologists in determining the appropriate treatment approach.

MOT contains immature teratomas (IT) as well as malignant transformation of mature cystic teratomas (MT-MCT) (6, 8). Nevertheless, no research has previously explored the connection between primary tumor laterality and prognostic outcomes among MOT patients. As such, further studies are warranted to examine how the primary tumor laterality influences the prognosis of MOT, to advance the development of personalized treatment approaches.

In this retrospective analysis, we examined MOT patients over a decade to explore the relationship between the primary tumor site and patient prognosis, both within the overall MOT population and when classified by histopathological subtypes.

## 2 Methods

### 2.1 Study population

In this retrospective study, patients with MOT were enrolled from The Affiliated Hospital of Qingdao University from January 2012 to December 2021. Patients with any of the following characteristics were excluded: 1) not diagnosed by the histopathological pathology; 2) with overlapping primary tumor sites or missing data on the primary tumor site; 3) with missing data on follow-up time or survival status. All patients provided written informed consent under the approval of the ethics committee of The Affiliated Hospital of Qingdao University (QYFY WZLL28449). All ethical standards, including ethics committee approval and the informed consent process, adhered to international guidelines.

### 2.2 Data collection

Part of the data collection process is similar to that of an article previously published (9). Clinical data for MOT cases were retrospectively obtained from the institutional Hospital Information System (HIS) system. Cases demonstrating IT or MT-MCT on histopathological review underwent expanded data collection, including patients’ information, clinical presentation, pathological characteristics, surgical outcomes, adjuvant therapies and survival outcomes. All histological specimens underwent blinded dual review by board-certified gynecologic pathologists, with concordance assessment through hematoxylin-eosin (HE) staining and immunohistochemistry for all included patients. A third reviewer participated in discussions to resolve any discrepancies. The standard tumor stage was defined by the International Federation of Gynecology and Obstetrics (FIGO) staging system in 2014. Primary endpoints were progression-free survival (PFS) and overall survival (OS). Progression-free survival (PFS) was defined as the time between the date of the first diagnosis and the date of ovarian cancer progression. Overall survival (OS) was defined as the time between the date of the first diagnosis and the date of death.

### 2.3 Statistical analysis

The groups’ baseline characteristics were compared using Chi-squared tests. To estimate survival curves for PFS and overall survival OS, Kaplan–Meier analysis was employed, and differences were evaluated using the log-rank test. Both univariate and multivariate Cox proportional hazards regression analyses were used to discern independent prognostic factors. Significant variables identified in the univariate analysis were incorporated into the multivariate analysis. The findings are expressed as hazard ratios (HRs) accompanied by 95% confidence intervals (CIs). Analyses were also conducted based on histopathological subtypes. All statistical evaluations were carried out with SPSS (version 28.0), and a two-sided *P*-value of less than 0.05 was deemed statistically significant.

## 3 Results

**Figure 1:**
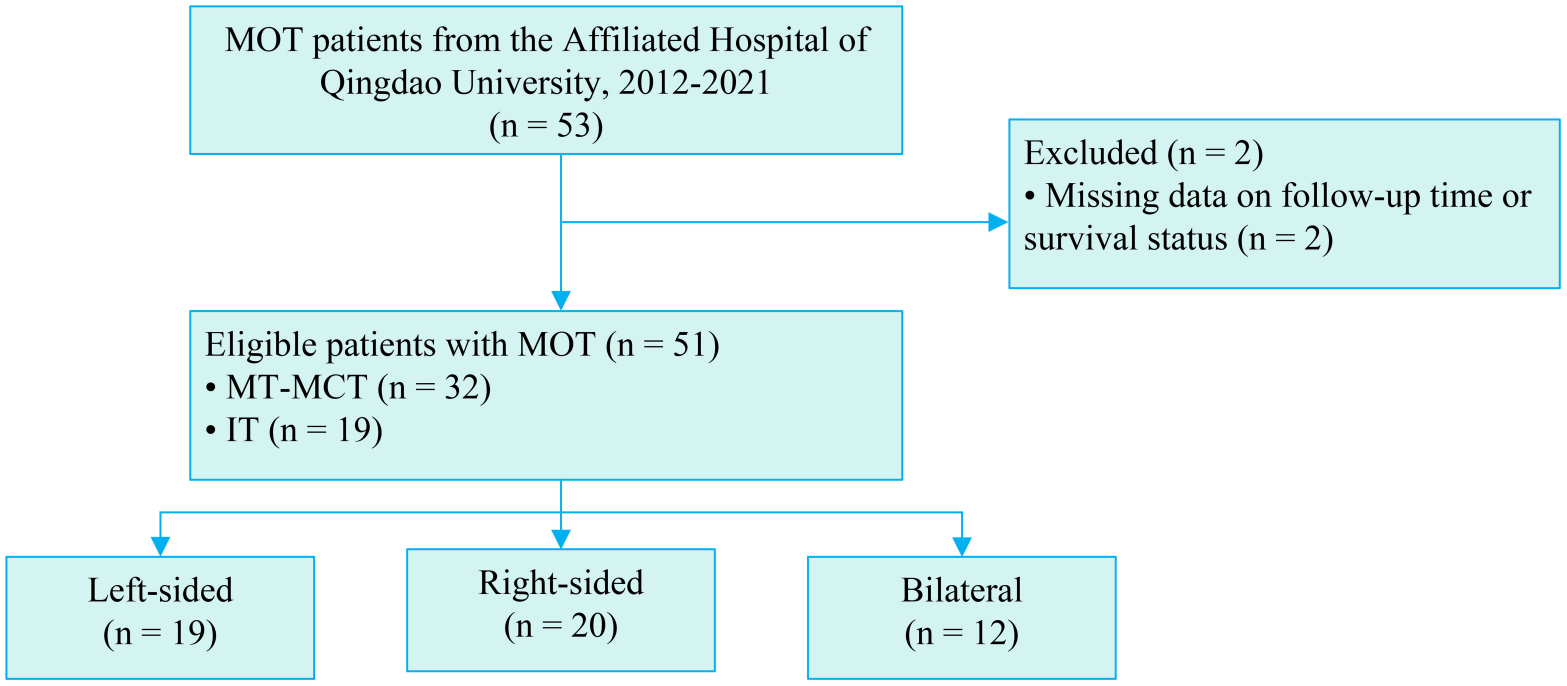
Flowchart of the study. MOT, malignant ovarian teratoma; MT-MCT, malignant transformation of mature ovarian teratoma; IT, immature teratoma.

A total of 53 women were diagnosed as MOT between 2012–2021 at The Affiliated Hospital of Qingdao University. Two patients were excluded because of missing data on follow-up time or survival status (**Figure 1**). Patient characteristics are summarized in **Table 1**. Thirty-two women were diagnosed with MT-MCT and nineteen were diagnosed with IT. The tumors in 19 women were located in the left ovary, 20 in the right ovary. The tumors of twelve women involved both ovaries. Patients’ age ranged from 10 to 78. The mean age of IT patients was 26, and the mean age of MT-MCT patients was 60. The median age of the total patients was 52 years. The mean diameter of all the tumors is 15 cm. MT-MCT predominated in bilateral cases (75.0%), while IT was more common in left-sided tumors (47.4%). Patients aged ≥52 years constituted 54.9% overall but were particularly prevalent in right-sided (55.0%) and bilateral tumors (75.0%); younger patients (<52 years) were more frequent in left-sided tumors (57.9%). Larger tumors (≥15 cm) were more common in left-sided (57.9%), while smaller tumors (<15 cm) predominated right-sided cases (70.0%). Bilateral tumors showed aggressive features: 75.0% were poorly differentiated (G3) and presented as FIGO Stage III. Abdominal pain/bloating was the most frequent symptom overall (56.9%) and universal in bilateral cases; 27.5% were asymptomatic, primarily right-sided tumors (45.0%). Fertility-sparing surgery was performed more often in left-sided tumors (68.4%). Normal serum AFP (≤20 ng/ml) was observed in 66.7% of patients overall, with elevated levels (>20 ng/ml) in 19.6%.

**Table 1:** Baseline characteristics of patients with MOT according to primary tumor laterality.

Characteristics	Overall (n=51)	Left-sided (n=19)	Right-sided (n=20)	Bilateral (n=12)	*P*-value
Histopathological type					<0.001
MT-MCT	32(62.7)	10(52.6)	13(65.0)	9(75.0)	
IT	19(37.3)	9(47.4)	7(35.0)	3(25.0)	
Age (yr)					<0.001
<52	23(45.1)	11(57.9)	9(45.0)	3(25.0)	
≥52	28(54.9)	8(42.1)	11(55.0)	9(75.0)	
Tumor size (cm)					<0.001
<15	28(54.9)	8(42.1)	14(70.0)	6(50.0)	
≥15	23(45.1)	11(57.9)	6(30.0)	6(50.0)	
Differentiated grade					<0.001
G1	23(45.1)	10(52.6)	10(50.0)	3(25.0)	
G2	10(19.6)	3(15.8)	7(35.0)	0	
G3	18(35.3)	6(31.6)	3(15.0)	9(75.0)	
FIGO stage					<0.001
I	30(58.8)	14(73.7)	16(80.0)	0	
II	10(19.6)	4(21.0)	3(15.0)	3(25.0)	
III	11(21.6)	1(5.3)	1(5.0)	9(75.0)	
IV	0	0	0	0	
Initial Symptoms					<0.001
Pelvic mass	4(7.8)	3(15.8)	1(5.0)	0	
Abdominal pain or bloating	29(56.9)	9(47.3)	8(40.0)	12(100.0)	
Poor appetite	1(2.0)	1(5.3)	0	0	
Malaise	1(1.9)	0	1(5.0)	0	
Increased abdominal girth	2(3.9)	1(5.3)	1(5.0)	0	
None	14(27.5)	5(26.3)	9(45.0)	0	
Time of diagnosis					<0.001
Pre-operation	6(11.8)	1(5.3)	2(10.0)	3(25.0)	
Intra- and post-operation	45(88.2)	18(94.7)	18(90.0)	9(75.0)	
BMI					<0.001
<18.5	1(2.0)	1(5.3)	0	0	
18.5-24.9	31(60.8)	12(63.2)	13(65.0)	6(50.0)	
≥25	19(37.3)	6(31.5)	7(35.0)	6(50.0)	
Surgical method					<0.001
Fertility-sparing	22(43.1)	13(68.4)	6(30.0)	3(25.0)	
Non-fertility-sparing	29(56.9)	6(31.6)	14(70.0)	9(75.0)	
Serum tumor marker (AFP)					0.244
≤20 ng/ml	34(66.7)	14(73.7)	14(70.0)	6(50.0)	
>20 ng/ml	10(19.6)	3(15.8)	4(20.0)	3(25.0)	
Missing	7(13.7)	2 (10.5)	2 (10.0)	3(25.0)	

MOT, malignant ovarian teratoma; MT-MCT, malignant transformation of mature cystic teratoma; IT, immature teratoma; FIGO, the International Federation of Gynecology and Obstetrics; BMI, body mass index; G1, well differentiated; G2, moderately differentiated; G3, poorly differentiated or undifferentiated.

**Figure 2:**
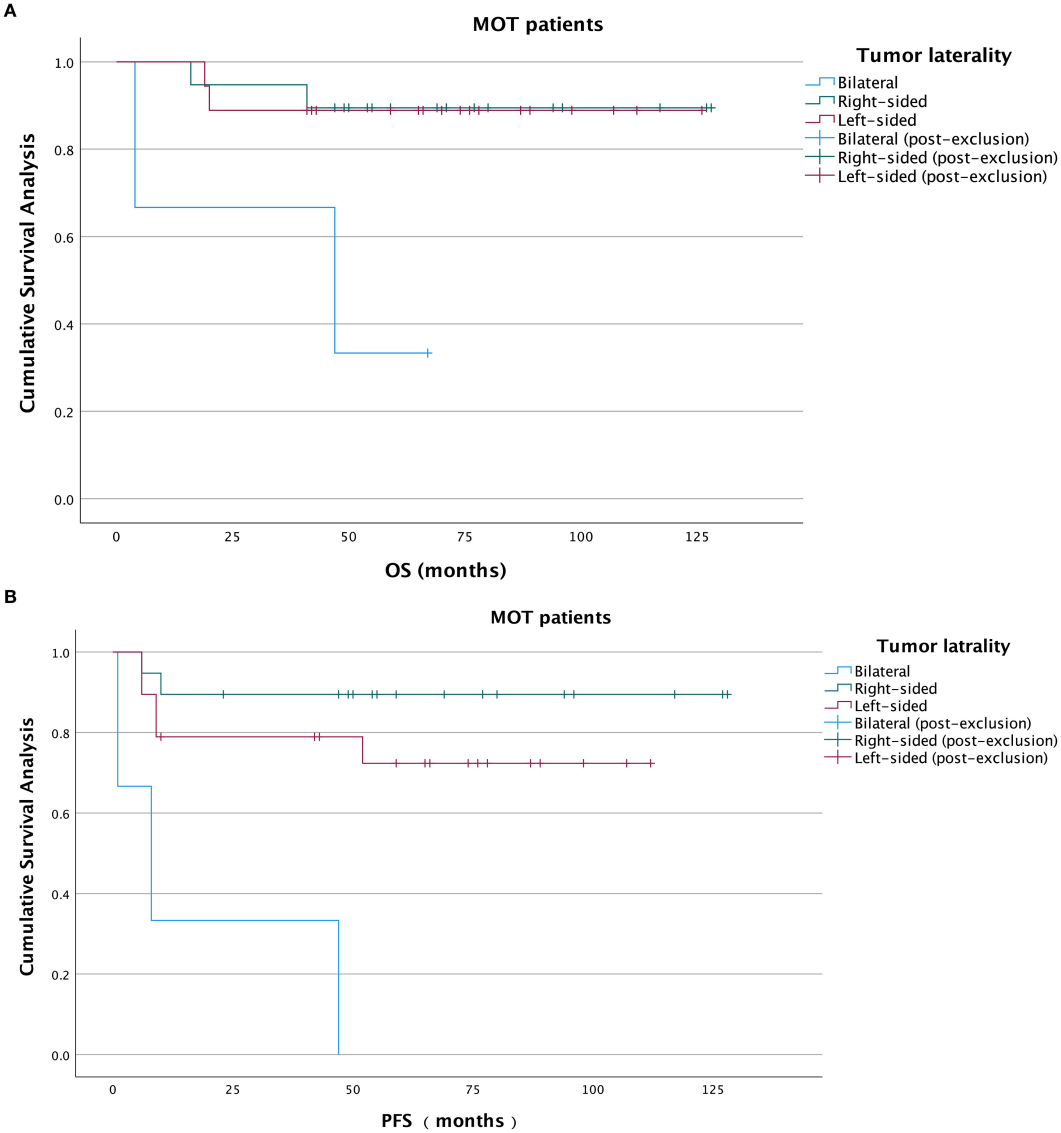
Kaplan-Meier analysis of OS and PFS in total MOT patients. OS curves in total patients **(A)**, PFS curves in total patients **(B)**. PFS, progression-free survival; IT, immature teratoma; MOT, malignant ovarian teratoma.

**Figure 3:**
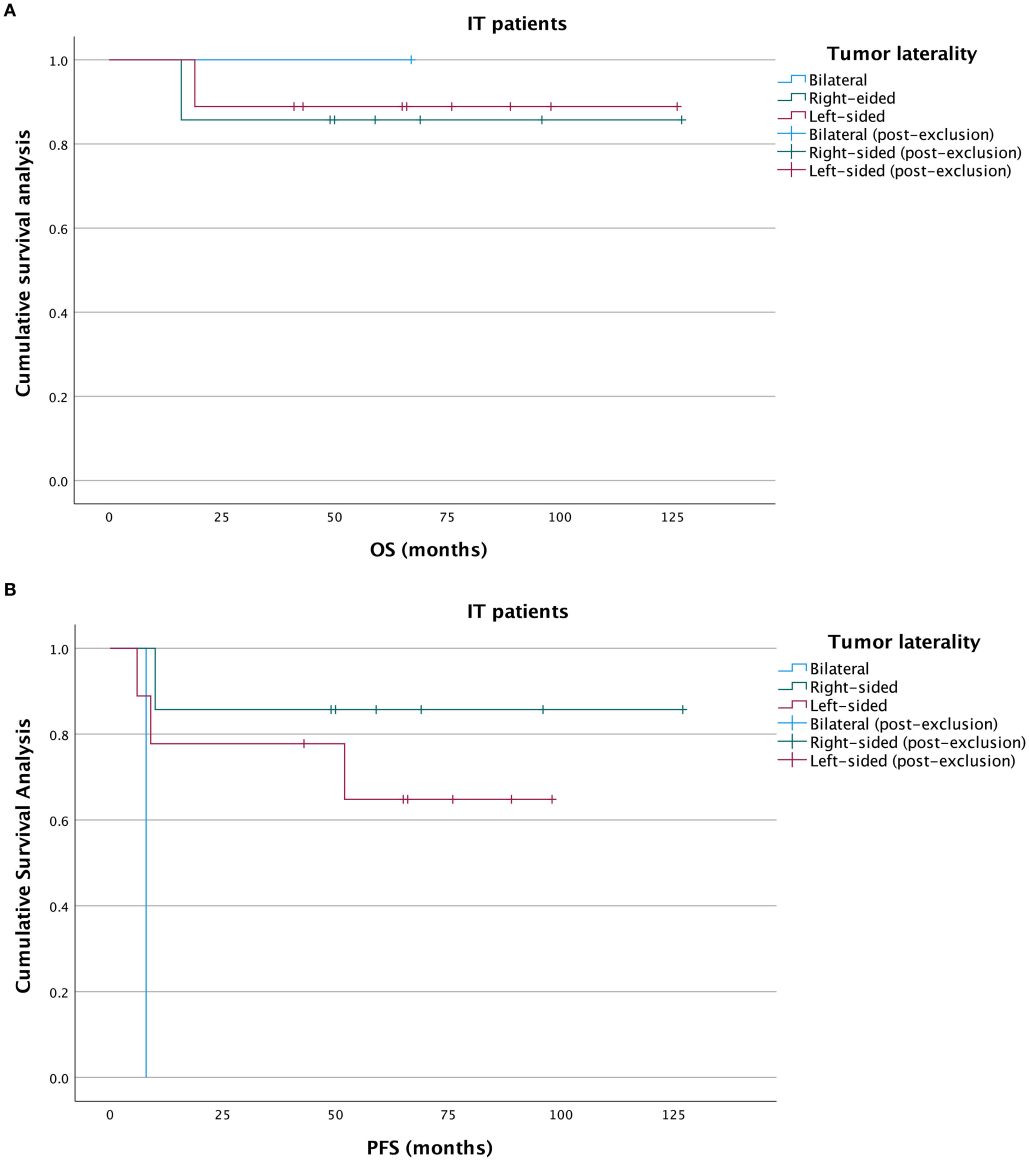
Kaplan-Meier analysis of OS and PFS in IT patients. OS curves in IT patients **(A)**, PFS curves in IT patients **(B)**. OS, overall survival; PFS, progression-free survival; IT, immature teratoma.

**Figure 4:**
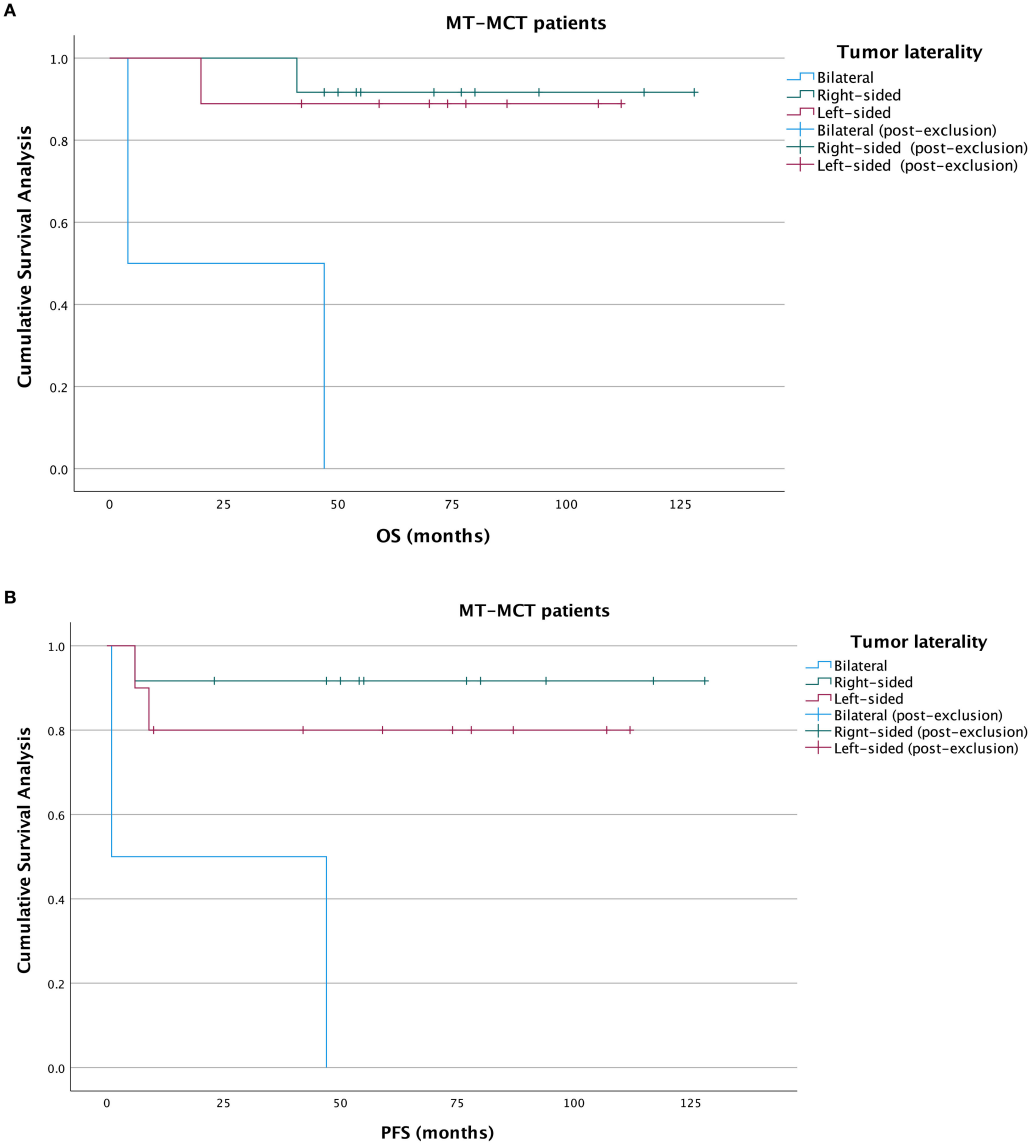
Kaplan-Meier analysis of OS and PFS in MT-MCT patients. OS curves in MT-MCT patients **(A)**, PFS curves in MT-MCT patients **(B)**. OS, overall survival; PFS, progression-free survival; MT-MCT, malignant transformation of mature ovarian teratoma.

With a median overall survival of 66.5 months, the 5-year OS rates in left, right-sided and bilateral groups were 84.2%, 85.0%, and 25%, respectively. The median PFS was 52 months. In the Kaplan-Meier analysis, OS was significantly poorer in the bilateral MOT group compared with the left-sided MOT group (*P* = 0.005) and the right-sided MOT group (*P* = 0.002). No significant difference in OS was observed between the left-sided and right-sided groups (P = 0.954) (**Figure 2A**). For PFS, the bilateral MOT group also showed significantly worse outcomes than both the left-sided MOT group (*P* < 0.001) and the right-sided MOT group (*P* < 0.001) (**Figure 2B**). However, among patients diagnosed with IT, no significant difference in OS was found between these three groups (**Figure 3A**). Notably, both the right-sided group (*P* = 0.003) and the left-sided group demonstrated significantly improved PFS (**Figure 3B**). In the MT-MCT group, no significant differences in OS or PFS were observed between the left-sided and right-sided groups. Bilateral MT-MCT patients had worse PFS and OS than left and right-sided groups patients (**Figures 4A, B**).

The univariate and multivariate Cox regression analyses showed that right-sided tumors (HR = 0.01; 95% CI, 0.01-0.04; *P* = 0.03) were associated with better PFS, compared to left-sided disease and bilateral disease. However, histopathological subtypes, grade, age, tumor size, chemotherapy and surgery methods were not independent prognostic factors (*P* > 0.05) (**Table 2**).

**Table 2:** Univariate and multivariate analysis of prognostic factors in MOT.

Characteristics	Univariate analysis			Multivariate analysis		
	HR	95%CI	*P*-value	HR	95%CI	*P*-value
Tumor laterality						
Left-sided	Ref					
Right-sided	0.37	0.07-1.89	0.23	0.01	0.00-0.04	0.03
Bilateral	7.09	0.01-0.94	<0.01	0.01	0.00-1.25	0.05
Histopathological type						
IT	Ref					
MT-MCT	0.11	0.41-2.99	0.83			
Age (yr)	1.02	0.99-1.05	0.14			
Tumor size (cm)						
<15	Ref					
≥15	1.03	0.39-2.74	0.96			
Differentiated grade						
G1	Ref					
G2	0.13	0.03-0.48	0.02	0.00	0-2.88×10^110	0.22
G3	0.42	0.13-1.41	0.16	0.10	0.01-1.22	0.60
FIGO stage						
I	Ref					
II	0.06	0.01-0.25	<0.01	12.37	0.00-12.68	0.84
III	0.48	0.15-1.66	0.19	11.57	0.00-41.78	0.20
BMI						
<18.5	Ref					
18.5-24.9	0.00	0.00-10.00	0.98			
≥25	1.26	0.44-3.63	0.67			
Surgical method						
Fertility-sparing	Ref					
Non-fertility-sparing	0.84	0.31-2.25	0.73			
Serum tumor marker (AFP)						
Missing	Ref					
≤20 ng/ml	1.25	0.23-6.86	0.79			
>20 ng/ml	0.76	0.24-2.43	0.65			

HR, hazard ratio; CI, confidence interval.

## 4 Discussion

In this study, patients with right-sided MOT exhibited significantly better prognosis than those with left-sided tumors, particularly among patients with MT-MCT. Additionally, patients with unilateral ovarian tumors appeared to have a more favorable prognosis compared to those with bilateral disease. Tumor laterality was associated with disease progression in MOT patients. However, no significant difference in overall survival (OS) was observed across the IT cohort.

Due to the different anatomical locations of tumors, the prognosis of malignant tumors originating from the same organ can vary, as observed in many solid tumors. However, data on the prognostic value of primary tumor laterality in MOT remain limited. In 1977, data from the National Cancer Institute indicated that women with bilateral ovarian cancer had lower survival rates than those with unilateral ovarian cancer (10). In 2011, a retrospective study by Mahdi et al. enrolled 1,529 patients with ovarian germ cell tumors, including 665 with MOT (11). They reported that bilateral MOT was also associated with poorer survival outcomes. In 2021, a cross-sectional study by He et al. examined the lateral distribution and related clinical characteristics of Chinese patients with benign ovarian teratomas (12). Their findings showed that recurrent ovarian teratomas occurred more frequently on the left side than on the right.

MOT patients showed varied PFS. Specifically, patients in the right-sided group had a 99% decreased risk of recurrence, compared to those in the left-sided group. These findings are consistent with the results of Zhang et al., who reported the impact of laterality on the prognosis of epithelial ovarian cancer (11). Cox regression analysis showed no significant difference between left-sided, right-sided and bilateral groups in both MT-MCT and IT patients, respectively. Due to the rarity of MOT, identifying prognostic factors has proven challenging, and relevant data remain scarce. Our findings provide new insights into the prognostic factors of MOT, which are crucial for understanding this rare malignancy in clinical practice.

The pathophysiological mechanisms underlying the different prognostic effects of laterality in MOT remain unclear. Anatomical disparities between the left and right hemipelvis may contribute to this phenomenon through their influence on metastasis patterns. Specifically, hematogenous dissemination represents a key route for distant spread in ovarian cancer. The distinct venous drainage pathways—where the right ovarian vein drains directly into the inferior vena cava, while the left drains into the left renal vein (13)—could facilitate more efficient systemic circulation of tumor cells from right-sided lesions. Conversely, the close anatomical proximity between the sigmoid colon and the left adnexa (14, 15) might promote direct tumor spread and local implantation on the left side. These asymmetric anatomical factors potentially explain the observed poorer prognosis associated with left-sided MOT.

In our study, tumor laterality was the only dependent prognostic factor. However, factors such as FIGO stage, age, histopathological subtypes and differentiated grade, have been regarded as significant prognostic factors for ovarian cancer in previous studies (16). Ovarian germ cell tumors frequently occur in children, particularly in cases of mature and immature teratomas (17), whereas MT-MCT typically arises in older women. This study included both younger and adult women, with the mean age of patients with IM being lower than that of those with MT-MCT. The prognosis for IT was also more favorable compared to MT-MCT. However, due to the small sample size, it remains unclear whether patient age at onset is an independent prognostic factor. Studies with larger sample size are needed to further assess the association between these factors and prognosis in patients with MOT.

This study’s primary strength lies in the novel exploration of the relationship between primary tumor laterality and prognosis in MOT. Using both Kaplan–Meier and Cox regression analyses, complemented by in-depth subgroup analyses, we were able to enhance the robustness and reliability of our findings. These results suggest potential clinical implications that merit further investigation. To our knowledge, we are the first to highlight the prognostic value of primary tumor laterality in MOT, offering a new perspective that could eventually help gynecological oncologists refine risk stratification for MOT patients pending validation in larger cohorts. This finding, if confirmed, could inform the future development of more personalized treatment strategies and follow-up plans.

Given the observed poorer prognosis in patients with left-sided or bilateral MOT in this study, future research could explore whether more intensive therapeutic approaches might be beneficial to reduce recurrence and improve survival outcomes. Conversely, the more favorable prognosis associated with right-sided MOT raises the question of whether less intensive treatment regimens and monitoring could be sufficient, potentially reducing the burden on both patients and healthcare systems. However, these treatment considerations are currently hypothetical and require robust clinical validation. Moreover, exploring the treatment response differences between MOT patients with different tumor laterality could offer valuable insights for developing more targeted and personalized therapeutic approaches.

Our study has several limitations. First, as a single-institution, retrospective study, it is prone to inherent selection bias. Second, the MOT group included a heterogeneous mix of histopathological subtypes, which may have distinct prognostic outcomes. Additionally, due to the rarity of MOT, the sample size was insufficient to thoroughly examine the relationship between primary tumor laterality and prognosis. Larger-scale prospective studies are needed to confirm these findings and explore the underlying biological mechanisms.

## 5 Conclusion

This retrospective study identified primary tumor laterality as an independent prognostic factor in MOT. Patients with right-sided MOT had the most favorable outcomes, while those with bilateral MOT experienced the poorest prognosis. Left-sided MOT patients exhibited an intermediate prognosis. Gynecologic oncologists could consider primary tumor laterality as a potential factor when assessing prognosis in MOT, and future studies should investigate whether incorporating laterality can optimize treatment and surveillance strategies.

## Data Availability

The raw data supporting the conclusions of this article will be made available by the authors, without undue reservation.
